# Prone Positioning in Moderate to Severe Acute Respiratory Distress Syndrome due to COVID-19: A Cohort Study and Analysis of Physiology

**DOI:** 10.21203/rs.3.rs-56281/v1

**Published:** 2020-08-17

**Authors:** Mehdi Shelhamer, Paul D. Wesson, Ian L. Solari, Deanna L. Jensen, William Alex Steele, Vihren G. Dimitrov, John Daniel Kelly, Shazia Aziz, Victor Perez Gutierrez, Eric Vittinghoff, Kevin K. Chung, Vidya P. Menon, Herman A. Ambris, Sanjiv M. Baxi

**Affiliations:** NYC Health and Hospitals Lincoln; University of California San Francisco; US Air Force Medical Service; US Air Force Medical Service; US Air Force Medical Service; NYC Health and Hospitals Lincoln; University of California San Francisco; NYC Health and Hospitals Lincoln; NYC Health and Hospitals Lincoln; University of California San Francisco; Uniformed Services University of the Health Sciences F Edward Hebert School of Medicine; NYC Health and Hospitals Lincoln; NYC Health and Hospitals Lincoln; University of California San Francisco

**Keywords:** coronavirus disease 2019, acute respiratory distress syndrome, prone position, severe acute respiratory syndrome coronavirus 2, respiratory failure

## Abstract

**BACKGROUND:**

Coronavirus disease 2019 (COVID-19) can lead to acute respiratory distress syndrome (ARDS) but it is unknown whether prone positioning improves outcomes in mechanically ventilated patients with moderate to severe ARDS due to COVID-19.

**METHODS:**

A cohort study at a New York City hospital at the peak of the early pandemic in the United States, under crisis conditions. The aim was to determine the benefit of prone positioning in mechanically ventilated patients with ARDS due to COVID-19. The primary outcome was in-hospital death. Secondary outcomes included changes in physiologic parameters. Fine-Gray competing risks models with stabilized inverse probability treatment weighting (sIPTW) were used to determine the effect of prone positioning on outcomes. In addition, linear mixed effects models (LMM) were used to assess changes in physiology with prone positioning.

**RESULTS:**

Out of 335 participants who were intubated and mechanically ventilated, 62 underwent prone positioning, 199 met prone positioning criteria and served as controls and 74 were excluded. The intervention and control groups were similar at baseline. In multivariate-adjusted competing risks models with sIPTW, prone positioning was significantly associated with reduced mortality (SHR 0.61, 95% CI 0.46–0.80, *P<* 0.005). Using LMM to evaluate the impact of positioning maneuvers on physiological parameters, the oxygenation-saturation index was significantly improved during days 1–3 (*P*< 0.01) whereas oxygenation-saturation index (OSI), oxygenation-index (OI) and arterial oxygen partial pressure to fractional inspired oxygen (P_a_O_2_:FiO_2_) were significantly improved during days 4–7 (*P*< 0.05 for all).

**CONCLUSIONS:**

Prone positioning in patients with moderate to severe ARDS due to COVID-19 is associated with reduced mortality and improved physiologic parameters. One in-hospital death could be averted for every eight patients treated. Replicating results and scaling the intervention are important, but prone positioning may represented an additional therapeutic option in patients with ARDS due to COVID-19.

## Background

Severe acute respiratory syndrome coronavirus 2 (SARS-CoV-2), the cause of coronavirus disease 2019 (COVID-19), has had a profound impact on global public health. The ongoing COVID-19 pandemic has presented numerous clinical management challenges further compounded by overwhelmed health systems. The initial critical care experience in Hubei province, and more broadly in China, inadequately informed preparations for what has been seen in Europe and North America.(1) Healthcare providers have therefore continued to incorporate and evaluate interventions in real-time. In the setting of critical COVID-19 illness, SARS-CoV-2 infection often results in severe pneumonia and hypoxemia with many patients developing acute respiratory distress syndrome (ARDS).(2) Hypoxemic respiratory failure with ARDS has poor outcomes overall and COVID-19 associated ARDS is no exception.(3, 4)

Several interventions for ARDS have been evaluated over the last two decades. In particular, prone positioning is one of few therapeutic interventions for patients with severe ARDS that has demonstrated improved oxygenation and a survival benefit.(5) Awake prone positioning outside of the intensive care unit (ICU) is safe and may decrease respiratory rate and improve oxygenation with early application potentially delaying need for intubation in patients with COVID-19.(6–8) In the ICU setting, prone positioning of patients receiving non-invasive ventilation or high-flow nasal canula, with or without sedation, may also be beneficial.(8) Physiologically, prone positioning may improve matching of ventilation and perfusion, but studies have not linked physiologic changes to clinical outcomes, especially in COVID-19.(9, 10)

The South Bronx is a socioeconomically disadvantaged borough in New York City (NYC) that had the highest per capita COVID-19 case count in the United States at 2941 per 100,000 residents with very high hospitalization and death rates.(11, 12) The pressing challenge that COVID-19 brought to NYC necessitated external support through the United States Departments of Defense and Homeland Security, re-distribution and up-training of local hospital staff, support from clinical volunteers, and augmentation through healthcare worker staffing agencies. Given the high volume of critically-ill patients admitted to the hospital, a multidisciplinary team was assembled to provide prone positioning given the support for the practice in other populations with ARDS.

We sought to determine if patients on mechanical ventilation with moderate to severe ARDS who underwent standardized prone positioning had lower mortality and improved within-person physiologic changes. As we rapidly evaluate drugs and interventions for COVID-19, it is crucial to understand if serial prone positioning could be a complementary therapeutic intervention for the most critically ill.

## Methods

### Study design

**Figure 1:**
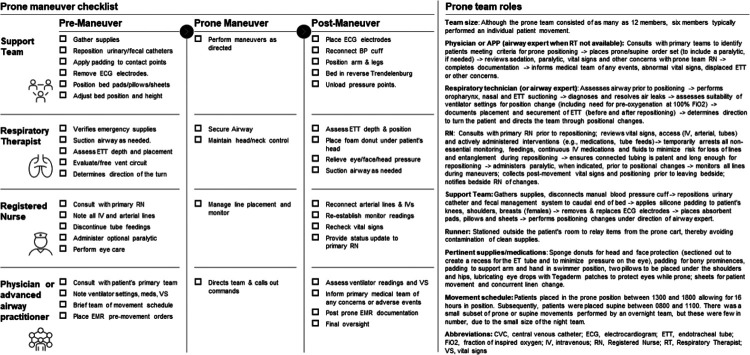
Prone team members, roles and checklists.

A cohort design with participants from the peak of hospitalizations for COVID-19 in exposed (prone positioning) and non-exposed (non-prone-positioning) groups. During the COVID-19 pandemic, much of the hospital was converted into make-shift intensive care units and virtually all inpatients had confirmed COVID-19. During this time, a multidisciplinary prone team including personnel from the United States Air Force Medical and Nursing Corps, the United States Army, civilian contractors, and hospital occupational and physical therapy was assembled to offer positioning maneuvers which were otherwise rarely done due to crisis operations. Details of the prone positioning process, including peri-maneuver checklists, team size and roles, supplies and team schedule are included in Fig. 1. In brief, patients were ideally put in the prone position in the afternoon allowing at least 16 hours in position before returning to supine position the following morning. The prone team included a physician, respiratory therapist, registered nurse, runner, and at least two members to safely support patient movements. The respiratory therapist served as the default airway expert except when a physician or advanced practice provider was trained in advanced airway management and, in that case, these providers served as airway expert.

### Setting and participants

Participants were identified from a single level 1 trauma hospital in the South Bronx, New York City, and were included across all hospital services (medicine, surgery, intensive care). All sequential adult patients (>17 years of age) were included if they were intubated, had not undergone prone positioning by others, met criteria for prone positioning, and had confirmed SARS-CoV-2 infection by real-time real-time reverse transcription-polymerase chain nasal swab (Bio-Reference Laboratories, Inc., Elmwood Park, NJ, USA) from March 25 through May 2, 2020. The prone team offered positional services for mechanically ventilated patients who met the following criteria (established *a priori*): arterial oxygen partial pressure to fractional inspired oxygen (PaO_2_:FiO_2_) <150 mm Hg, positive end-expiratory pressure (PEEP) >10 cm of water and FiO_2_ > 0.6. The ultimate decision for initiating and discontinuing positional movements was made by the primary team overseeing and coordinating care for each patient. Prone positioning was not mandatory, but was routinely available, 24 hours a day, seven days a week. The study received institutional review board approval (IRB # 20 – 007).

### Measures and outcomes

The primary exposure was positional maneuvers, defined as regular alternation between prone and supine positioning. The primary outcomes of interest were in-hospital mortality and, among exposed patients, differences in physiological parameters in prone vs supine position. In the mortality analysis, every patient had at least 30 days elapse following initiation of, or meeting criteria for, prone positioning. Follow-up of unexposed controls began when the participant first met prone positioning criteria during the two weeks after intubation. In the analysis of positioning effects on physiologic parameters among exposed patients, repeated measures of the oxygenation index (OI), oxygenation saturation index (OSI), PaO_2_:FiO_2_ and SpO_2_:FiO_2_ were compared during periods of prone and supine positioning. Episodes of positioning separated by more than 48 hours were considered separately. The last physiologic measurement collected in the intervals between each positional change were used in the analysis. After the final positioning change, the last measurement collected within 24 hours was used. Confounders for both analyses were identified based on literature review and directed acyclic graphs. In particular, age, sex, race, body mass index (BMI), acute physiology and chronic health evaluation (APACHE-II) score and vasopressor use were the primary confounders by indication. In the mortality analysis, the APACHE-II score was evaluated at the time of intubation.(13) BMI and age were categorized for ease of interpretation and clinical utility. The study team obtained the study data through manual electronic medical record chart abstraction (Epic Systems, Verona, WI, USA).

### Statistical analysis

Characteristics of the cohort were summarized using descriptive statistics as appropriate. Fine-Gray models were used to assess the association between prone positioning and death, accounting for hospital discharge as a competing risk.(14) Participants remaining in the hospital at the end of follow-up were censored. The proportional sub-distribution hazards assumption was assessed visually through cumulative incidence curves. To minimize confounding by indication, we used standard regression adjustment as well as a doubly robust approach adding stabilized inverse probability treatment weights (IPTWs) to the fully adjusted model.(15–17) A sensitivity analysis was done to identify changes in results by excluding controls that died within 48 hours of intubation. In addition, number needed to treat was calculated by the inverse of the averaged absolute risk differences at 30 days, for all participants at their actual and counterfactual values of prone positioning, and in combination with their observed covariate values.(18)

Linear mixed models (LMMs) were used to assess the association of prone vs supine positioning with physiologic parameter levels among the exposed. Outcomes were natural log transformed to meet normality assumptions. The LMMs included nested random effects for participant and positioning episode, and allowed for autocorrelation of the residuals. In addition to estimating overall positional effects, we also estimated these effects in days 1–3 and 4–7 of each episode. Pearson correlation coefficients were also used to characterize degree of agreement for OI, OSI, PaO2:FiO2 and SpO2:FiO2, to support clinical utility in practice. Analyses were performed in Stata (Version 16, StataCorp, College Station, TX, USA).

## Results

**Figure 2:**
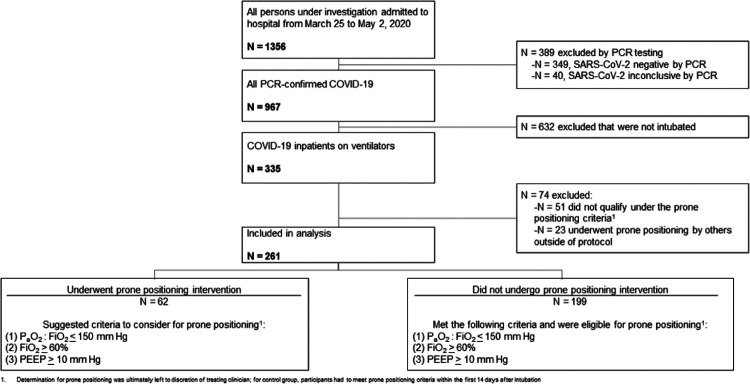
Determination of prone positioning groups during intervention period.

During the study period, 335 individuals were intubated and placed on mechanical ventilation. Sixty-two underwent prone positioning while 199 who did not undergo positioning changes, but met criteria to do so, were selected as contemporary controls. Seventy-four individuals were excluded for failing to meeting prone positioning criteria or for having undergone prone positioning by providers outside of the standard protocol. A study flow diagram depicts the inclusion and exclusion of participants across groups in Fig. 2. Overall, study participants were older, male and mostly self-reported Hispanic or Black. The majority of participants were obese. Diabetes and obstructive lung disease were the most common comorbidities. Most patients were critically ill and septic on admission with a median APACHE-II score at intubation of 17. Most participants required mechanical ventilation at hospital admission (i.e., intubated in the emergency room) and almost all patients (85%) received at least some amount of hydroxychloroquine as was consistent with hospital policy during the time. Most patients ultimately expired within two weeks. Compared to the control group, the participants who underwent prone positioning were younger (60 versus 66 years old) and were more frequently classified as severe rather than critical on admission to the hospital. Proportions of sepsis on admission and median APACHE-II scores at the time of intubation were similar across groups, but the prone positioning intervention group had less ARDS on admission. Full baseline, demographic and outcome data is summarized in Table 1.

### Prone positioning and mortality

**Figure 3:**
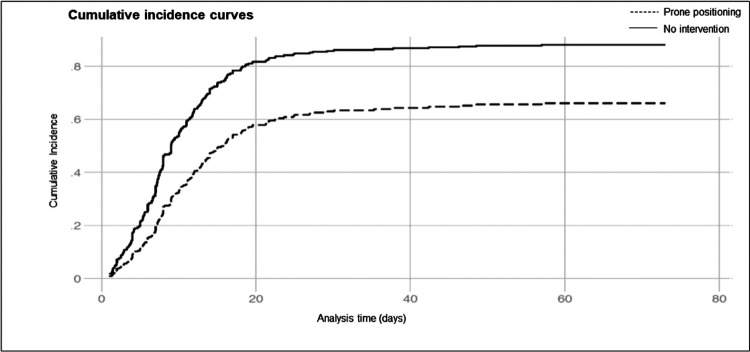
Cumulative incidence curves for participants undergoing prone positioning versus not.

Compared to contemporary controls, the prone positioning group had fewer deaths and a longer time to death in those who expired, in spite of similar length of stay and ventilator-free days. Estimates of the association between prone positioning and mortality are summarized in Table 2. Unadjusted and adjusted competing risks analysis showed that exposed patients were at reduced risk of death (SHR 0.51, 95% CI 0.39–0.66, p< 0.005 and SHR 0.57, 95% CI 0.42–0.76, p < 0.005, respectively) compared to unexposed controls. In the doubly-robust analysis adding stabilized IPTWs, inferences were similar (SHR 0.61, 95% CI 0.46–0.80, p < 0.005) and for every eight patients that underwent prone positioning, one in-hospital death was averted. We found no evidence for violation of the proportional hazards assumption through visual inspection of cumulative incidence curves (Fig. 3). Covariate effect estimates are available in Table 3. A sensitivity analysis with removal of controls who died within 48 hours of intubation (N = 18) showed similar results.

### Prone positioning and physiologic parameters

**Figure 4:**
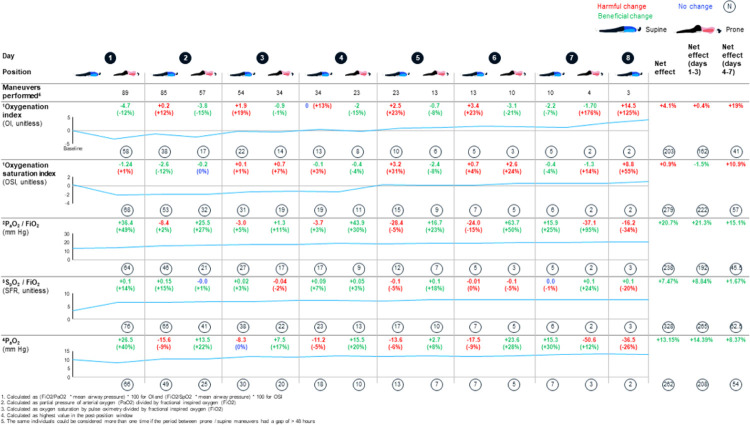
Within-person variability in mean physiologic parameters through prone positioning across days of the intervention.

**Figure 5:**
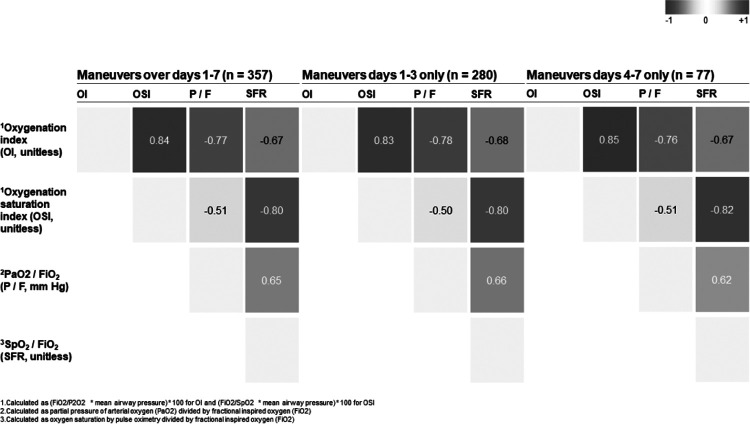
Pearson correlation of physiologic parameters across prone patients split by duration of maneuvers.

Figure 4 shows the mean trajectories of physiologic parameters over time. Improvements were seen for days 1–3 in the OSI, P_a_O_2_:FiO_2_, S_p_O_2_:FiO_2_ and P_a_O_2_. For days 4–7 of prone positioning, improvement was seen in the P_a_O_2_:FiO_2_, S_p_O_2_:FiO_2_ and P_a_O_2_. Only the OI failed to show improvement at any time and OSI did not show improvement for days 4–7. During crisis operations with enhanced infection control and use of transport ventilators for routine ventilation, it may be difficult to obtain P_a_O_2_ and mean airway pressure values and so proxy variables may be helpful. We therefore looked at Pearson correlation coefficients amongst ratios and indices. Overall, P_a_O_2_:FiO_2_ and S_p_O_2_:FiO_2_ are moderately correlated (*p* = −0.51), and OSI and OI, and OSI and S_p_O_2_:FiO_2_, are closely correlated (p = 0.84 and *p* = −0.80, respectively). The correlations did not differ when split into days 1–3 and 4–7. Results are summarized in Fig. 5.

In analyses using LMMs to estimate the association of positioning with physiological indices, 19 of 62 exposed participants contributed more than one episode. In these analyses, prone vs supine positioning was significantly associated with overall improvement in P_a_O_2_:FiO_2_ (Table 4). In models allowing positioning effects to differ in days 1–3 and 4–7, prone positioning was associated with improved OSI during days 1–3 (p < 0.01) as well as improved OSI, OI and P_a_O_2_:FiO_2_ during days 4–7 (p < 0.05, p < 0.01 and p< 0.001, respectively). No clear evidence for interaction between positioning and time was found.

## Discussion

We report results from a comprehensive cohort study assessing the potential benefits of prone positioning in COVID-19 patients with moderate to severe ARDS. We found a nearly 40% reduction in mortality with prone positioning, an effect that appears sustained on cumulative incidence curves. With respect to physiologic parameters, there were meaningful changes across all ratios and indices to suggest that prone positioning is associated with improvements in within-person physiology and that the benefit may persist beyond three days. Our findings across both analyses were robust to various adjustments, modifications, sensitivity analyses and nested comparative testing.

Fundamentally, this study has three key findings. First, we demonstrated a mortality benefit with prone positioning with a number needed to treat of eight. The durability of the finding is important, and ensuring that it can be replicated in other settings will be essential to justify a recommendation, but we have no evidence to attribute the survival benefit in the intervention arm to bias. Second, it appears that there is a benefit to additional days of prone positioning beyond 3 days. The effect seen with 4–7 days of prone positioning may be heavily influenced by a smaller group that realized a differential benefit, but 34 of 89 positioning sequences resulted in at least four days of intervention, representing a relatively large proportion of individuals. Third, prone positioning resulted in significant changes in physiologic parameters which may support the underlying hypothesis that prone positioning improves ventilation-perfusion matching.(9, 10) Additionally, we demonstrated the utility of relatively accessible clinical information in the ICU as reasonable surrogates to monitor changes in physiology.

Our results are consistent with recent multi-center data suggesting a mortality benefit of prone positioning in patients with ARDS whether intubated or not.(6–8, 19, 20) There are recommendations for prolonged prone positioning of 12–16 hours daily for mechanically ventilated adult patients with COVID-19 and refractory hypoxemic respiratory failure,(21) but the optimal duration of the intervention, its’ impact on physiologic parameters and details regarding how to organize and structure an intervention team during a crisis have not been completely evaluated. We acknowledge that prone positioning in mechanically ventilated patients is a resource-intensive intervention, particularly in overwhelmed healthcare systems during pandemic conditions. Before adopting prone positioning techniques, staff education and commitment is paramount. If justified by hospitalized patient volume, we recommend identifying personnel and assigning them to a dedicated prone team and tailoring readily available checklists to institutional needs and constraints (Fig. 1).(22)

Some limitations of this study should be noted. First, this is a single center retrospective cohort study in a resource constrained environment under crisis operations. As a result, although patients had critical care needs, they were frequently cared for in ad-hoc intensive care units by non-critical care personnel. The decision to initiate or discontinue the intervention under study was left to the treating primary team without defining endpoints. We attempted to address any residual confounding through IPTW and no differences in the results were noted. If the prone team was consulted and the patient had moderate to severe ARDS and met criteria for prone positioning, it was felt that they could benefit from the intervention in addition to lung protective ventilation. Although this was pragmatic for this setting, if prone positioning is implemented elsewhere, the prone teams could consider establishing an opt-out approach with tailored entry and exit criteria, normal cadence of evaluation for candidacy for prone positioning and a mechanism for real-time data capture and quality control assessments. Finally, the results may not be readily generalizable to all populations, in particular those with milder disease and those that don’t reflect the ethnic diversity seen in the Bronx. The institutional mortality proportion was high (>75%) and therefore the impact of the intervention may be attenuated in the setting of advanced interventions (e.g., extracorporeal membrane oxygenation).

There are also some notable strengths of this work. We were able to collect detailed physiologic data in a structured manner to systematically evaluate the impact of the intervention. Also, our population has been gravely understudied in the COVID-19 pandemic and we’ve been able to contribute significantly to both describing their clinical course as well as critical care interventions for socioeconomically marginalized minority populations. Regarding outcome, we were able to include all patients who would have been eligible for prone positioning as controls creating a sound counterfactual for a contemporaneous comparison of both exposed and unexposed. Finally, compared to existing literature for patients with COVID-19, this study provides results for a large intervention group.

## Conclusions

In summary, we present data supporting prone positioning as an intervention to prolong survival and improve physiologic parameters in patients on mechanical ventilation with moderate to severe ARDS due to COVID-19. The findings should be replicated across institutions, but prone positioning may be an important consideration for health systems, particularly in the setting of an evolving suite of complementary interventions in the care of such vulnerable patients.

## Figures and Tables

**Table 1:** Baseline characteristics, including demographic and clinical presentation and outcomes, for all participants in the prone positioning intervention and non-prone positioning groups.

	Overall	Underwent prone positioning	Did not undergo prone positioning
	n = 261	n = 62	n = 199
Age, years (median, IQR)	64.0 (55.0–73.0)	60.0 (54.3–66.5)	66.0 (55.0–74.5)
Age (years), No. (%)			
< 41 years	13 (5.0%)	3 (4.8%)	10 (5.0%)
41–60 years	85 (32.6%)	27 (43.5%)	58 (29.1%)
61–80 years	131 (50.2%)	31 (50.0%)	100 (50.3%)
> 80 years	32 (12.3%)	1 (1.6%)	31 (15.6%)
Sex, female, No. (%)	99 (37.9%)	20 (32.3%)	79 (39.7%)
Race, No. (%)			
Hispanic	170 (65.1%)	38 (61.3%)	132 (66.3%)
Black	63 (24.1%)	12 (19.4%)	51 (25.6%)
Asian	2 (0.8%)	0	2 (1.0%)
White	6 (2.3%)	0	6 (3.0%)
Other	20 (7.7%)	12 (19.4%)	8 (4.0%)
Body mass index, kg/m^2^ (median, IQR)	31.0 (27.1–6.8)	30.9 (28.3–35.9)	31.0 (26.7–37.2)
Body mass index, No. (%)			
< 18.5 kg/m^2^	3 (1.1%)	0	3 (1.5%)
18.5–24.9 kg/m^2^	33 (12.6%)	5 (8.1%)	28 (14.1%)
25–29.9 kg/m^2^	78 (29.9%)	19 (30.6%)	59 (29.6%)
≥ 30 kg/m^2^	147 (56.3%)	38 (61.3%)	109 (54.8%)
**Clinical symptoms on presentation, No. (%)**			
			
Fever	159 (60.9%)	41 (66.1%)	118 (59.3%)
Cough	190 (72.8%)	54 (87.1%)	136 (68.3%)
Shortness of breath	220 (84.3%)	54 (87.1%)	166 (83.4%)
GI symptoms (diarrhea or vomiting)	36 (13.8%)	12 (19.4%)	24 (12.1%)
Neurological symptoms (altered mental status or seizures)	55 (21.1%)	5 (8.1%)	50 (25.1%)
**Comorbidities, No. (%)**			
Current smoking	14 (5.4%)	1 (1.6%)	13 (6.5%)
Diabetes	127 (48.7%)	27 (43.5%)	100 (50.3%)
Obstructive lung disease (asthma or COPD)	54 (20.7%)	10 (16.1%)	44 (22.1%)
Congestive heart failure	19 (7.3%)	1 (1.6%)	18 (9.0%)
Autoimmune disease (RA or SLE)	15 (5.7%)	3 (4.8%)	12 (6.0%)
Chronic kidney disease (Stage ≥ 3)	29 (11.1%)	4 (6.5%)	25 (12.6%)
latrogenic immunosuppression	6 (2.3%)	1 (1.6%)	5 (2.5%)
Cancer	17 (6.5%)	2 (3.2%)	15 (7.5%)
Human immunodeficiency virus infection	5 (1.9%)	2 (3.2%)	3 (1.5%)
Renal Transplantation	3 (1.1%)	1 (1.6%)	2 (1.0%)
Charlson Comorbidity Index (median, IQR)	3.0 (2.0–4.0)	3.0 (1.0–4.0)	3.0 (2.0–5.0)
**Severity of COVID-19 on admission, No. (%)** (13, 23)			
Moderate	11 (4.2%)	6 (9.7%)	5 (2.5%)
Severe	86 (33.0%)	27 (43.5%)	59 (29.6%)
Critical	163 (62.5%)	29 (46.8%)	135 (67.8%)
APACHE-II score (median, IQR) at intubation	17.0 (12.0–27.0)	a17.5 (12.3–24.0)	17.0 (12.0–28.0)
ARDS on admission	146 (55.9%)	27 (43.5%)	119 (59.8%)
Sepsis on admission by Quick SOFA	160 (61.3%)	38 (61.3%)	122 (61.3%)
**Radiological characteristics, No. (%)**			
Bilateral reticulonodular opacities	173 (66.3%)	41 (66.1%)	132 (66.3%)
Ground-glass opacities	96 (36.8%)	28 (45.2%)	68 (34.2%)
Focal consolidation	31 (11.9%)	5 (8.1%)	26 (13.1%)
**Treatment and clinical course, No. (%)**			
BiPAP prior to mechanical ventilation	37 (14.2%)	17 (27.4%)	20 (10.1%)
Mechanical ventilation on admission	186 (71.3%)	31 (50.0%)	155 (77.9%)
Vasopressor use during hospital course	221 (84.7%)	53 (85.5%)	168 (84.4%)
Acute kidney injury during hospital course	142 (54.4%)	29 (46.8%)	113 (56.8%)
Hemodialysis required during hospital course	35 (13.4%)	16 (25.8%)	19 (9.5%)
Hydroxychloroquine administered	219 (83.9%)	52 (83.9%)	167 (83.9%)
**Maneuvers and adjustments**			
Total maneuvers	-	832	-
Prone positioning	-	199	-
Supine positioning	-	190	-
Head, neck and shoulder adjustments	-	443	-
Maneuvers per participant (median, IQR)	-	4 (2–8)	-
**Outcomes (followed minimum of 30 days), no (%)**			
Expired	215 (82.4%)	48 (77.4%)	167 (83.9%)
Discharged	43 (16.4%)	13 (21.0%)	30 (15.1%)
Ongoing hospitalization	3 (1.1%)	1 (1.6%)	2 (2.0%)
Time to death (median, IQR) from admission	8.2 (5.4–13.5)	15.3 (12.2–21.7)	7.2 (4.2–10.9)
Length of stay, days (median, IQR)	9.0 (5.4–14.3)	18.1 (13.1–26.9)	8.0 (5.0–14.0)
Ventilator-free days (median, IQR)	18.0 (13.0–22.0)	19.0 (16.0–20.0)	18.0 (12.0–22.0)
Total extubations	29 (11.1%)	7 (11.3%)	22 (11.1%)
Total re-intubations	8 (3.1%)	1 (1.6%)	7 (3.5%)
Palliative extubations	10 (3.8%)	2 (3.2%)	8 (4.0%)
Tracheostomy	26 (10.0%)	13 (21.0%)	13 (6.5%)
**Laboratory values on admission, [reference range and units] reported as median (IQR), N reported if different from total**			
White blood cell count [4.8–10.8 × 10^3^ microliter]	9.5 (6.9–12.9)	9.5 (7.1–12.6)	9.6 (6.8–13.1)
Platelet count [150 to 450 per microliter]	235 (182–301)	211.5 (186–283)	237.0 (181–303)
Highest d-dimer during hospital course [< = 230 ng/milliliter]	3543 (1163–11838), n = 218	3988 (2049.5–13049.8)	3185 (1064–11739), n = 156
C-reactive protein [0–0.40 mg/deciliter]	28.0 (14.8–100.0), n = 244	24.1 (14.3–35.9), n = 61	30.8 (15.7–122.2), n = 183
Highest creatinine during hospital course [0.7–1.20 mg/deciliter]	3.7 (1.5–6.9), n = 260	3.8 (1.1–6.6)	3.7 (1.7–7.1), n = 198
Lactate [0.5–2.2 mmol/liter]	2.1 (1.4–3.2), n = 223	2.0 (1.5–3.2), n = 56	2.1 (1.4–3.2), n = 167
Procalcitonin [< = 0.08 ng/milliliter]	0.5 (0.2–1.3), n = 230	0.5 (0.3–1.3), n = 55	0.5 (0.2–1.3), n = 174
lnterleukin-6 (0–5.5 pg/milliliter)	19.8 (15.2–251.3), n = 220	16.1 (15.0–150.7), n = 57	32.3 (15.2–273.5), n = 162
Ferritin [20–250 ng/milliliter]	928.5 (515–1625), n = 225	871.0 (487–1466), n = 59	949 (531–1670), n = 166
International normalized ratio [0.8 to 1.1]	1.3 (1.1–1.4), n = 240	1.3 (1.2–1.4), n = 59	1.3 (1.1–1.4), n = 181

ARDS, acute respiratory distress syndrome; BiPAP bilevel positive airway pressure; COPD, chronic obstructive pulmonary disease; Pro-BNP-N-terminal pro b-type natriuretic peptide; RA, rheumatoid arthritis; SLE, systemic lupus erythematosus

**Table 2:** Association of a prone positioning intervention and time to death by Fine-Gray competing risks analysis.

Model	SHR	95% CI	*P*-value
Unadjusted	0.51	0.39–0.66	< 0.005
Multivariate adjusted	0.57	0.42–0.76	< 0.005
Stabilized doubly robust IPTW	0.61	0.46–0.80	< 0.005

Adjusted models control for age, sex, race, body-mass index, Apache II score and vasopressor use

**Table 3:** Complete modeling output for Cox regression, with inverse-probability treatment weighting, adjustments, stabilized weights and accounting for competing risks.

Variable	SHR	95% CI	*P*-value
Prone positioning intervention (yes vs no)			
No	Reference	-	-
Yes	0.61	0.46–0.80	< 0.001
Age			
< 41 years	Reference	-	-
41–60 years	2.68	0.83–8.59	0.10
61–80 years	4.45	1.39–14.20	0.01
> 80 years	7.11	2.13–23.76	0.001
Sex			
Female	Reference	-	-
Male	1.06	0.78–1.44	0.69
Race			
White	Reference	-	-
Hispanic	0.33	0.18–0.60	< 0.001
Black	0.38	0.20–0.73	0.003
Asian	*	*	*
Other	0.34	0.12–0.96	0.04
Body mass index, No. (%)			
< 18.5kg/m^2^	*	*	*
18.5–24.9 kg/m^2^	Reference	-	-
25–29.9 kg/m^2^	0.85	0.53–1.36	0.49
≥ 30 kg/m^2^	0.87	0.57–1.33	0.52
APACHE-II score	1.01	0.99–1.03	0.26
Vasopressor use			
No	Reference	-	-
Yes	1.18	0.76–1.85	0.46

* Observations were dropped from model due to small N and no variability in treatment (e.g. all within category were treated or all within category were not treated)

**Table 4:** Adjusted associations of prone vs supine positioning with physiological parameters by linear mixed effects models.

	Oxygenation index	Oxygenation saturation index	PaO2:FiO2	SpO2:FiO2
Number (maneuvers)	N = 59 (85)	N = 60 ()	N = 59 ()	N = 54 (76)
	Coefficient (95% CI)	Coefficient (95% CI)	Coefficient (95% CI)	Coefficient (95% CI)
Prone, overall	0.07 (−0.01, 0.2)	0.04 (−0.01, 0.08)	0.10 (0.04, 0.17)	−0.28 (−0.63, 0.08)
% *improvement*	*8%*	*4%*	*11%*	*24%*
Prone days 1–3	0.1 (−0.1, 0.1)	0.08 (0.0, 0.1)**	0.05 (−0.0, 0.1)	−0.32 (−0.7, 0.01)
% *improvement*	*1%*	*8%*	*5%*	*27%*
Prone days 4–7	0.30 (0.1, 0.5)**	−0.10 (−0.2, 0.0)	0.31 (0.2, 0.5)***	−0.03 (−1.0, 0.9)
% *improvement*	*38%*	*(9% worsening)*	36%***	*3%*
Days 4–7 vs 1–3	−0.08 (−0.2, 0.1)	0.09 (−0.0, 0.2)	−0.8 (−0.2, 0.1)	0.06 (−0.7, 0.8)

Adjusted for age, sex, race, BMI, Apache II score, and vasopressor use

* *P*< 0.05,

** *P*< 0.01

*** *P*< 0.001

## Abbreviations

ARDS: Acute Respiratory Distress Syndrome
APACHE-II: Acute Physiology And Chronic Health Evaluation
BMI: Body Mass Index
COVID-19: Coronavirus Disease 2019
FiO_2_: Fraction of Inspired Oxygen
ICU: Intensive Care Unit
IPTWs: Inverse Probability Treatment Weights
LMM: Linear Mixed Models
NYC: New York City
OI: Oxygenation Index
OSI: Oxygenation-Saturation Index
PaO_2_: Partial Pressure of Arterial Oxygen
PEEP: Positive End-Expiratory Pressure
SARS-Cov-2: Severe Acute Respiratory Syndrome Coronavirus 2
sIPTW: Inverse Probability of Treatment Weight
SPO_2_: Oxygen Saturation
